# Overweight and Obesity Epidemic in Developing Countries: A Problem with Diet, Physical Activity, or Socioeconomic Status?

**DOI:** 10.1155/2014/964236

**Published:** 2014-10-14

**Authors:** Trishnee Bhurosy, Rajesh Jeewon

**Affiliations:** Department of Health Sciences, Faculty of Science, University of Mauritius, Réduit, Mauritius

## Abstract

Obesity is a significant public health concern affecting more than half a billion people worldwide. Obesity rise is not only limited to developed countries, but to developing nations as well. This paper aims to compare the mean body mass index trends in the World Health Organisation- (WHO-) categorised regions since 1980 to 2008 and secondly to appraise how socioeconomic disparities can lead to differences in obesity and physical activity level across developing nations. Taking into account past and current BMI trends, it is anticipated that obesity will continue to take a significant ascent, as observed by the sharp increase from 1999 to 2008. Gender differences in BMI will continue to be as apparent, that is, women showing a higher BMI trend than men. In the coming years, the maximum mean BMI in more developed countries might be exceeded by those in less developed ones. Rather than focusing on obesity at the individual level, the immediate environment of the obese individual to broader socioeconomic contexts should be targeted. Most importantly, incentives at several organisational levels, the media, and educational institutions along with changes in food policies will need to be provided to low-income populations.

## 1. Introduction

According to the World Health Organisation [WHO] Media centre [1], in 2008, a total of more than half a billion adults were obese worldwide. The worldwide prevalence has more than doubled since 1980. A number of studies have reported that with each surge in weight, there is an increase in the risks for coronary heart disease, type 2 diabetes, cancers (endometrial, breast, and colon), hypertension, dyslipidaemia, stroke, sleep apnea, respiratory problems, osteoarthritis, and gynaecological problems [menstrual irregularities and infertility] [2].

During the last 30 years, demographic, economic development, environmental, and cultural changes have been impressive, particularly from 1970 to 1999, in developing regions [3]. During this period, a continuous reduction in underweight with a simultaneous increase in obesity has been reported [3]. In the United States (US) alone, medical costs related to obesity have risen from $78.5 billion a year in 1998 to $147 billion annually in 2008 [4]. If current trends in obesity prevail, total healthcare costs attributable to obesity could reach up to the range of $861 to $957 billion by 2030 in the US [5]. The case of the US can be referred to as an example to demonstrate the unestimated costs related to obesity in developing countries. Indirect costs are projected to be as much as *₤*27 billion ($45 billion) by 2015 [6]. Expressed as a percent of the total Gross Domestic Product, total health care costs associated with obesity in China have been twice as much as in the US and in India; obesity-related costs have been almost the same as in the US [7].

Changes in diet for the past 30 years have been significant in terms of more fat, more meat, added sugars and bigger portion sizes. “Nutrition transition,” termed as a combination of improved access to food, decreased physical activity level (PAL) has been identified to be the prime risk factor for the increasing prevalence of overweight and chronic metabolic diseases in the developing countries [8]. Such dietary patterns' alterations are often the manifestations of societal and environmental changes that emerge as a result of a lack of supportive policies in health, agricultural, transport, urban planning, food processing, distribution, marketing, and educational sectors [1].

Initially, such dietary shifts and the emergence of obesity were primarily related to the higher socioeconomic (SE) strata of the populations among developing countries [9]. However, more recent trends demonstrate a shift in the prevalence from the higher to the lower socioeconomic level [9]. For instance, to date, in Mauritius (middle-income country), recent studies indicate clearly that obesity is on the rise on several target populations, namely, among middle-aged [10] and postmenopausal women [11] and adolescents of low SES [12]. Notably, low level of education and moderate PAL, cost per calorie, and weight of food items are important mediators identified in the SES-BMI relationship [10, 12].

However, the number of studies conducted in developing countries to assess the obesity-socioeconomic status (SES) relationship is minimal [13]. Moreover, discrepancies in studies can also be attributed to the use of a single or a combination of SE indicators as each SE variable has its own strengths and drawbacks when linked to BMI [14]. Thus, understanding the concomitant outcomes of various SES indicators could yield important etiological insights into the SES-obesity relationship among developing countries [15]. Most importantly, there have been no studies on the comparison of the BMI trends among distinct WHO-categorised regions as from 1980. Thus, the objectives of this review are to

- compare the mean body mass index trends in WHO-categorised countries since 1980 to 2008;
- document how socioeconomic disparities can lead to differences in obesity and physical activity level across developing nations.

## 2. Obesity Outlook Worldwide

Past and ongoing studies indicate that during the last 30 years, there are significant changes in mean body weight, diet, and physical activity taking place along with progressive economic development in developing countries. It is highly probable that obesity and its comorbidities will continue to affect an increasing number of populations in these regions. Lifestyle and environmental factors are acting in a synergistic manner to fuel the obesity epidemic.

**Figure 1:**
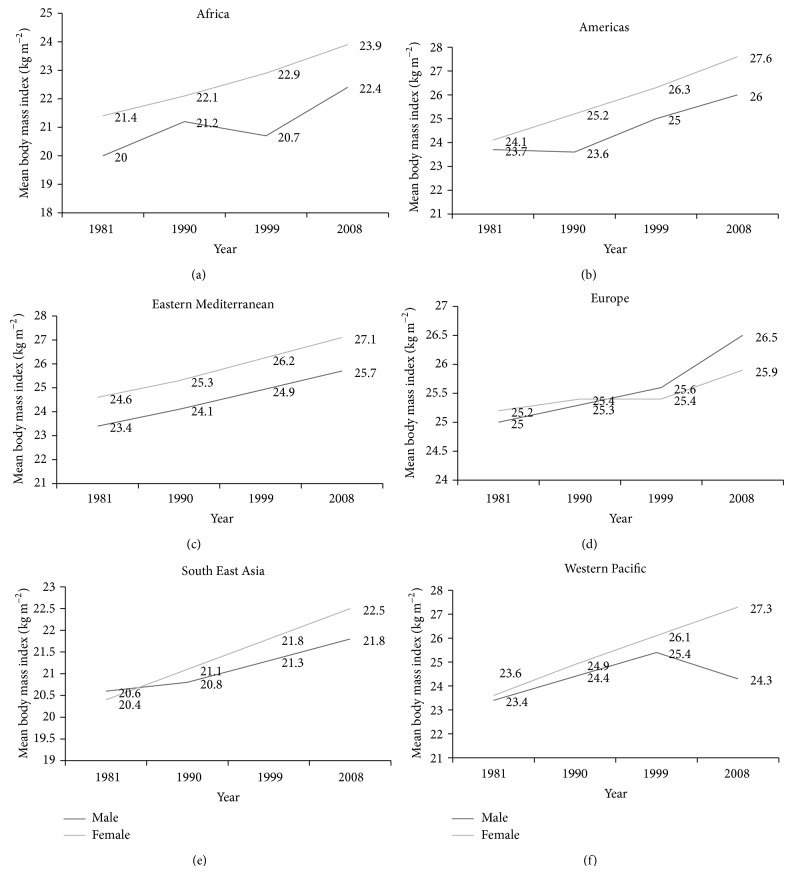
(a) Mean body mass index trends (age-standardised estimate) in Africa. (b) Mean body mass index trends (age-standardised estimate) in America. (c) Mean body mass index trends (age-standardised estimate) in Eastern Mediterranean. (d) Mean body mass index trends (age-standardised estimate) in Europe. (e) Mean body mass index trends (age-standardised estimate) in South East Asia. (f) Mean body mass index trends (age-standardised estimate) in Western Pacific.

In this study, we relied on data which was available on mean body mass index (BMI) values of the six WHO-categorised regions, namely, Africa, The Americas, Eastern Mediterranean, Europe, South-East Asia, and Western Pacific [16]. BMI values were reported by the WHO up to 2008. BMI has been universally employed to assess a person's weight status and health with regards to obesity. An average of BMI for each WHO-categorised region during four particular years (1981, 1990, 1999, and 2008) was then calculated. Each consecutive year shared a 10-year gap inclusive, for instance, from 1981 to 1990. Figures 1(a) to 1(f) illustrate the BMI (age-standardised estimate) from 1981, 1990, and 1999 to 2008 in WHO-categorised regions.

From 1981 to 1990, 1990 to 1999, and 1999 to 2008 inclusive and also from 1981 to 2008, for both men and women, a paired sample *t*-test was conducted to compare the differences in mean adult body mass index (BMI) over the span of 10 years. For men, a significant difference in mean BMI was noted from the year 1981 to 1990 while for women, a significant difference in mean BMI was observed for each range of years (*P* < 0.01). From 1981 to 2008, for both genders, mean BMI increased significantly [male: 24.4 (1.97) kgm^−2^ and female: 25.7 (2.08) kgm^−2^] (Table 1).

The figures and Table 1 show that, in general, the prevalence of obesity has been increasing since 1981 for both genders. More particularly, in the years 1999–2008, a sharp increase in BMI can be observed for all regions, except for a decline in male BMI in the Western Pacific (Figure 1(f)). However, lower mean BMI does not necessarily imply lowered risk of obesity as, for example, in Singapore (Western Pacific country), a higher percentage of body fat was reported at lower levels of BMI when compared to Caucasians [17]. This relationship holds true for Asians as well [18]. Findings of past cross-sectional and prospective epidemiological studies do suggest that the WHO BMI cut-offs do not adequately represent the obesity status worldwide [18]. Thus, it has been suggested that for distinct ethnic groups and for the pediatric population also, the use of waist circumference (WC) might be a better alternative since WC is a specific measure of truncal obesity along with being a strong predictor of visceral obesity [19]. Another noteworthy finding is that with the exception of European countries, female BMI was greater than that of males for every range of years (Figure 1(d)). Epidemiological studies suggest that women are more likely to store fat subcutaneously than men [20]. However, confounding factors like occupational differences between males and females, higher recreational physical activity among men, dietary pattern, and reproductive diversities among the two genders might have led to discrepant findings [21].

Adult mean BMI levels of 20.0 to 23.0 kg/m^2^ are predominant in Africa and South East Asia while levels of 24.0 to 27.0 kg/m^2^ are mostly prevalent across America, Europe, Eastern Mediterranean, and the Western Pacific regions. Though lower BMI trends are more prevalent in African and South East Asian countries, it has been forecast that developing nations will soon face the levels of overweight currently prevalent in USA and Europe [21]. Even countries in the Middle East and North Africa such as Egypt and Kuwait have already exceeded these levels [21].

Taking into consideration the past and current BMI trends, it is anticipated that, undoubtedly, obesity will continue to take a significant ascent. Oncoming data collected as from 2009 and onwards by the WHO will show similar trends in BMI as observed by the sharp increase from 1999 to 2008 for most regions. Gender differences in BMI will also continue to be as apparent as it is currently, that is, women showing a higher BMI trend than men. The mean BMI levels reported in Africa and South East Asia (20.0–23.0 kg/m^2^) will soon level those of developed regions and it is anticipated that in the coming years, the maximum mean BMI in more developed countries might be exceeded by those in less developed ones.

## 3. Socioeconomic Status and Obesity in Developing Countries

Since the early 1980s, economic globalisation in developing countries has driven changes in dietary patterns and food choices. Since food choice is mainly dictated by its price in the developing world, eliciting the influences of socioeconomic variables on food choice may be useful in explaining food behavior. Rapidly growing, developing, or transitional economies face the globalisation of food markets, fast food chains, and the increasing availability of street vendors who offer products at very competitive value due to economical acquisition of inputs such as raw and processed foods [22]. Differences in diet quality arise due to more frequent consumption of fresh and better quality produce such as fresh fruits, vegetables, and fish among higher socioeconomic status (SES) individuals since fresh produce items are charged higher in grocery and convenience stores [23]. In particular, the poorer segments are often left to opt for energy-dense diets, rich in cheap vegetable oils, and trans-fats [14]. For example, in countries of the Middle-East, Asia, and Africa, edible oil consumption has risen very rapidly [23]. In addition, the price per weight of food items is an important determinant of food choice. Low fat protein sources, for example, poultry and pulses, which cost less per weight, are the preferred choices of low SES participants [13].

In every country worldwide, whether transitional economies or developed ones, noncommunicable chronic conditions like obesity are either on the rise or have already reached alarming levels [24]. While low socioeconomic status (SES) has been associated with a higher prevalence of obesity and chronic diseases in developed countries, previous studies, in developing nations, have shown a positive SES-obesity relationship [25]. More recently, the SES-obesity relationship in developing countries has been reported to bear similarities to that in developed ones.

**Figure 2:**
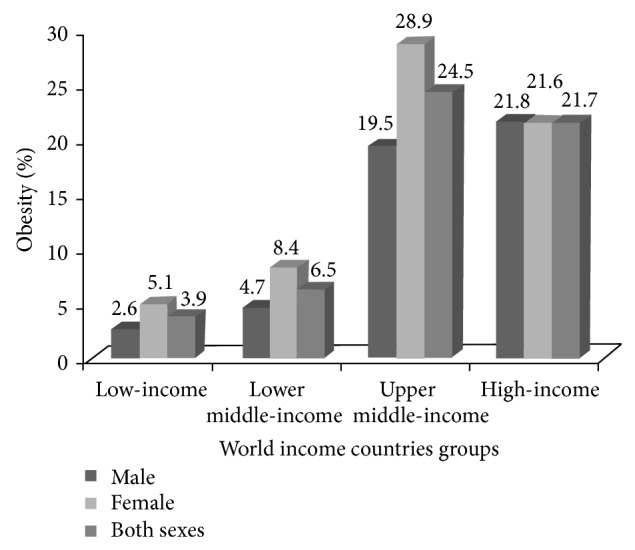
The obesity prevalence among adults aged ≥20 years by World Bank income groups in 2008 (%). Source: Statistical data taken from World Health Organisation [14].

As per Figure 2, with increasing income level of countries, the prevalence of obesity increases up to upper middle-income countries. The prevalence of obesity among women (28.9%) and in both genders (24.5%) is also the highest in upper middle-income countries, followed by high-income nations. Female obesity is higher than male obesity for all categories of income countries, except in the case of high-income countries whereby male obesity (21.8%) is slightly more elevated than that for females (21.6%). Women in high-income countries favour a leaner body image and, hence, engage themselves in higher physical activity to remain fit [26].

In light of current evidence, it can be predicted that the obesity pandemic will be unabated in the near future and low-income and lower-income countries will face the current trends of obesity observed in the upper-middle and high-income countries in the coming years. In fact, education is the socioeconomic indicator which has been reported to be the most significant predictor of diet quality [27]. Likewise, in Mauritius, a study conducted among young and middle-aged women [11] demonstrated that educational level was the only factor significantly associated with diet quality. Educational disparities reflect educational differences pertaining to dietary knowledge, food purchasing behaviour, and perceptions of healthy food items.

## 4. Physical Activity Level and Its Impact on Obesity: Current Scenario in Developing Countries

Low physical activity level (PAL) accounts for 6% of deaths worldwide and inadequate PAL, especially, concerns populations of low SES [28]. Reductions in PAL, over the past years, are linked to several factors: less energy expenditure activities such as farming and forestry, a rise in sporadic activities such as sitting in front of a computer terminal, and patterns of low activity during leisure hours [29, 30]. As such, concurrent marked reductions in PAL have been reported within every occupation [23]. To illustrate the significant increase in mean BMI noted from 1981 to 1990 which may be associated with reduced PAL (Table 1), Africa stands as a proper example. During these years, in African regions, the epidemic of obesity, at least, can in part be explained by decreased levels of physical activity as in the late 1980s; roads were tarred with taxis and buses becoming the most common transport means and, in addition, there was an ongoing trend away from manual labour to less physically strenuous jobs and the shift to less nutrient-dense diets [31].

Use of screen time has been associated with other equally unhealthy behaviours such as eating palatable fatty foods [32]. Watching television is linked to high cholesterol levels and unhealthy diets and which is also influenced by unhealthy nutrition messages in commercials [33]. In addition, low PAL is amplified by inadequate community designs and infrastructure characteristics such as lack of safe walkways, bicycle paths, and playgrounds [34].

Though low socioeconomic status and low educational level in developing countries are associated to low PAL due to, primarily, a worse access to sports facilities, PAL is twice as much among rural residents than urban ones due to higher household activities which compensate for low PAL during free time [28]. Since data on PAL is scarce and fragmented and is mostly based upon self-reports, information gathered on PAL in developing nations may be subjected to bias and the use of pedometers and other monitoring technologies is not yet widespread, even in developed countries [35].

### 4.1. Socioeconomic Disparities in Physical Activity Level

Despite its important role highlighted in the prevention of obesity, regular physical activity level (PAL) is low among many populations, more particularly among low socioeconomic status (SES) ones. For instance, daily moderate or vigorous PAL is undertaken by only 16.5% of Mauritians [36]. Hence, a better understanding of the causal relationships that underlie SES, PAL, and body mass index (BMI) is warranted to yield higher success rate of interventions.

Even if there are a small number of studies conducted on the SES-PAL relationship among adults, several plausible explanations have been suggested on how socioeconomic disparities may influence PAL, thus impacting on body weight or BMI. Firstly, there is a significant lack of resources available in medium- and low-SES neighbourhoods to practice sufficient PAL and of those few leisure-time PAL facilities available, the majority is not affordable by the lower SES groups [37]. In addition, as SES of a person increases, he/she becomes more health conscious and, therefore, this is reflected in his/her behaviour and BMI [24]. Also, higher SES people, especially women, are more likely to endorse health ideals such as more PAL to preserve a good body image [26]. Another plausible explanation is that even when low SES participants reported greater energy expenditures, they undertook less vigorous leisure physical activity than their high SES counterparts [38]. Another factor which has been less discussed in previous studies is a lack of self-esteem among low SES people who adopt less positive attitudes towards physical activity [39].

## 5. Relevance to Practice

Over the past years, initiatives have been doubled to mitigate obesity. However, these have been met with little or no success. Effectiveness of current intervention programs should, therefore, be reassessed and put into perspective so as to better tackle the rising prevalence of obesity. Referring to Mauritius, as an example, a national action plan for physical activity has been launched for the year 2011–2014 [36]. However, little data exists regarding its effectiveness on PAL in different regions in Mauritius. Published statistics among several target populations revealing elevated levels of overweight and obesity [10–12] inevitably calls into question the implementation of such a program. In fact, publishing a nutrition and physical activity plan is just the first step of many that a state must take as implementation and follow-through are the most important steps [40]. The United States Department of Agriculture concluded that despite many low SES individuals being recipients of food assistance programs, studies demonstrated that these programs have not lowered obesity [41].

Traditionally, obesity prevention is aimed at behavioural changes and lifestyle modification at a personal level and it is still the case today, leading to widespread stigma directed at obese individuals even by health professionals [42, 43]. Much time, money, and effort is risked into believing that obesity is a matter of personal responsibility while crucial opportunities to make key environmental changes and have a greater impact on obesity prevention are missed [44]. The situation is further exacerbated by different concepts of obesity prevention made available to the obese person through public health authorities, the food and marketing industry, and, lastly, the government [45]. Obese individuals are unable to make healthy choices when they are wrongly influenced towards unhealthy ones [41]. Concerns have also been raised over the use of BMI as an obesity indicator. Much bias may arise due to BMI variations arising as a result of ethnicity, age, sex, and differences in body build [45]. Other measures of fatness such as WC should be considered in conjunction with BMI to assess body-specific areas such as the location or distribution of fat in the abdomen.

A multifaceted approach to the treatment of obesity that addresses psychological, social, environmental, and biological factors is critical to ensure comprehensive care and best outcomes and practices [46]. Pragmatic ways for redefining success in the prevention of obesity have been proposed and suggested that obesity prevention should be focused on long-term improvement of health status and on small, but significant, changes in behaviour such as increased PAL and decreased fat intake [47].

While these suggested solutions on obesity prevention are true, the situation in developing countries demands the needs of several incentives at the level of the governments, nongovernmental organisation, and other equally important stakeholders such as the media. Relevant policies, nutrition education, and physical activity programmes, which will integrate these components reported by Robinson et al. [47], should be implemented on a large scale. At the same time, diet quality from manufacturing to sale should be rigorously monitored to ensure the equal and sufficient distribution of nutrient-dense foods to low-income populations.

## 6. Conclusions

The prevalence of obesity has been increasing since 1981 for both genders. Female BMI is greater than that of males, except in European countries. Lower BMI trends are more prevalent in African and Southeast Asian countries. As economic transition advances, it is predicted that the current mean BMI trends depicted in developing nations will surpass even the maximum mean BMI values reported in developed countries. Such an increase in overweight can be attributed to significant alterations in eating habits and physical activity level caused by socioeconomic influences. Rather than focusing on obesity as an individual behavioural chronic state, current scientific evidence indicates the consideration of a multidisciplinary approach targeting the immediate environment of the obese individual to broader socioeconomic contexts. For such a venture to be fruitful in developing nations, the incentives at several levels of organisations, the media, and the educational institutions along with changes in food policies and distribution will need to be provided to low-income populations.

## Figures and Tables

**Table 1:** Differences in mean BMI from 1981 to 2008 for both genders.

Year	Mean (SD) BMI kgm^−2^ (Male)	Statistical difference in BMI across years (*P* value)	Mean (SD) BMI kgm^−2^ (Female)	Statistical difference in BMI across years (*P* value)
1981	22.7 (1.95)	1981–1990: *P* < 0.05	23.2 (1.90)	1981–1990: *P* < 0.01
1990	23.2 (1.82)	1990–1999: *P* > 0.05	24.0 (1.89)	1990–1999: *P* < 0.01
1999	23.8 (2.20)	1999–2008: *P* > 0.05	24.8 (1.94)	1999–2008: *P* < 0.01
2008	24.4 (1.97)	1981–2008: *P* < 0.01	25.7 (2.08)	1981–2008: *P* < 0.01

BMI, body mass index; SD, standard deviation.

Statistical test: paired sample *t*-test.
